# The Impact of Faith-Based Pastoral Care in Decreasingly Religious Contexts: The Australian Chaplaincy Advantage in Critical Environments

**DOI:** 10.1007/s10943-023-01791-x

**Published:** 2023-03-28

**Authors:** Mark D. Layson, Lindsay. B. Carey, Megan C. Best

**Affiliations:** 1 Faculty of Arts and Education, St Mark’s National Theological Centre, Charles Sturt University, Canberra, ACT, NSW Australia; 2 Palliative Care Unit, La Trobe University, Melbourne, VIC Australia; 3 Institute for Ethics and Society, University of Notre Dame, Sydney, Australia; 4 Centre for Spirituality, Theology and Health, Duke University, North Carolina, USA; 5 School of Medicine, University of Sydney, New South Wales, Australia

**Keywords:** Chaplains, Military, Pastoral care, Religion, Secularism, Spiritual care, Moral injury, Critical environments, Bio-psycho-social-spiritual

## Abstract

This article considers the contribution of faith-based chaplains who provide holistic pastoral and spiritual care within critical environments such as the military, first responders, and hospitals. The contribution of faith-based chaplains can sometimes be taken for granted or not properly understood, particularly in some Western countries which are currently experiencing a decline in religiosity. Following on from a previous paper regarding chaplaincy utilization (Layson et al. 2022), this article presents an alternative argument to the secularist-humanist perspective by noting five ways by which the faith based chaplaincy model provides best practice service and builds a capability advantage for organizations that engage faith-based chaplaincy services. The first section discusses faith-based chaplaincy and organizational holistic care; the second section considers the role of faith-based chaplains—much of which is largely unknown and poorly appreciated; the third section considers the unique capability of faith-based chaplains to provide spiritual and religious care to those of faith and for those of none; the fourth section explores how faith-based chaplains can leverage the positive impact of religious organizations to provide additional low-cost resources for other organizations and their staff; and lastly, the operational advantage of faith-based chaplains on the world stage is considered, particularly in light of culturally and linguistically diverse populations to whom religiosity is increasingly important.

## Introduction

In recent years, the spiritual and religious landscape has been rapidly changing in most Western countries. For example in Australia, the number of Australians identifying with “no religion” has increased from 19% in 2006 to 30% in 2016 and 38.9% in 2021 (Australian Bureau of Statistics, 2022). Similarly in England and Wales “no religion” has increased from 25.2% in 2011 to 37.2% in 2021 (Office for National Statistics, 2022). The upward trend of “no religion” mirrors large decreases in Christianity; however, this is countered to a small degree by the current progressive increase in non-Christian religions. That is to say, for example, that while Christianity remains Australia’s most common religion (43.9%), the fastest growing religions are Hinduism (2.7%) and Islam (3.2%) (Australian Bureau of Statistics, 2022).

Indeed, while some argue that the world will become less religious (Inglehart, 2021), others predict that the world will become more religious (see Table 1; Pew Research Center, 2017). As our world changes, we can expect many Western countries, where secularism is currently the strongest, to become more culturally and religiously diverse. For example, between 2017 and 2021, while 28.6 per cent of new migrants to Australia had no religion, 29.1 per cent were Christian and 39.9 per cent were from other religions (Australian Bureau of Statistics, 2022). That is to say, despite the decline of Australian Christianity, Australia may well become more (rather than less) religious in future decades through migration and lower birth rates among the religiously unaffiliated (Pew Research Center, 2015). Yet, while informative, these official statistics only report the barest outlines of changes in belief structures.

**Table 1:** Projected growth of major religions by 2060 ^*^Source: Pew research centre (2017) demographic projections-the changing global religious landscape

Religious group	Projected 2060 population	Percentage of the world population in 2060
Christians	3,054,460,000	31.8
Muslims	2,987,390,000	31.0
Hindus	1,392,900,000	14.5
Unaffiliated	1,202,300,000	12.5
Buddhists	461,980,000	4.8
Folk religions	440,950,000	4.6
Other religions	59,410,000	0.6
Jews	16,370,000	0.2
World / Total	9,615,760,000	100

### Spirituality

While national statistical organizations measure affiliation with official religions, these statistics do not actually measure the broader category of spirituality. Amidst the decline in religiosity (noted above), faith and spirituality continue to be ongoing elements of Western cultures. A recent national Australian survey found that 68% of the population still have religious or spiritual beliefs and 55% discuss spirituality or religion “often” or “occasionally”, with younger Australians particularly interested in spirituality (McCrindle, 2017). An examination of secularism in America notes that while “religion” falls, and “no religion” rises, there is an ongoing interest in the supernatural and paranormal (Baker & Smith, 2015). Furthermore, as discussed later, the relatively recent decline of religiosity exists in a world which, nevertheless, remains overwhelmingly religious, and probably increasingly so (see Table 1; Pew Research Center, 2017). What this suggests is that, while there is a move away from having a specific affiliation with an official religion, the situation with regard to transcendent and supernatural beliefs, or the search for spirituality (meaning and purpose), is much more complex than the simple decline in religiosity statistics.

### “No Religion” Diversity

The situation with respect to those who have “no religious affiliation” makes analysing the contours of secularization complex and ambiguous. An international study of people who are atheists (i.e. people who “don’t believe in God”) and agnostic (i.e. people who “don’t know whether to believe there is a God or not”), found that there is significant diversity in how this group further identifies. Only about 2% identify as “secular” or “humanist” (Woodhead, 2017, p. 254) and approximately 90% of this cohort can fluctuate in affiliation over time (Lim et al., 2010, p. 22). This latter group constitute a dynamic cohort who are often difficult to accurately quantify or uniformly categorize.

### Secularist Exclusivity and Christian Inclusivity

Within the context of this complex religious and spiritual setting, some secular humanist organizations often campaign forthrightly against any religious input into the public square (Caro, 2022; National Secular Lobby, 2018). Specifically, some secularists (Hoglin, 2021a, 2021b; Savage, 2018; Surman, 2009) attempt to argue that faith-based welfare services, such as chaplaincy, are unable to fulfil pastoral/spiritual care for non-religious personnel. This argument is made despite numerous historical accounts detailing considerable chaplaincy work undertaken over many decades and which note, for example, how non-religious military personnel have had continuous engagement, respect and great affection for their “padres” (Davidson, 2021; Gladwin, 2013; Shay, 2014; Strong, 2012). Despite these historical accounts and the contemporary utilization and acceptance of faith-based chaplains, some secularists are advocating for the total exclusion of faith-based chaplaincy—which must be considered discriminatory, given that nearly all organizations (public and private) employ personnel with religious/spiritual beliefs.

For most organizations, removing faith-based services and simply having non-religious practitioners, would reduce the diversity of care and would in effect duplicate other secular services already provided (which will be noted again later). This duplication would in turn prove to be economically inefficient, as many organizations already offer, and widely promote, multiple secular well-being services via employee assistance programs (EAPs), medical doctors, nurses, psychologists, counsellors, mental health contractors, sexual assault personnel and/or social workers.

Others argue that faith-based well-being services, such as chaplaincy (which significantly pre-date other allied professions such as psychologists and social workers), have always provided care for both religious and non-religious people for centuries, caring for “those of all faiths and those of none” and they continue to do so (Carey, 2012; Cheboi, 2021). Indeed, the care offered to dying pagans by Christians during early Roman plagues changed the Western world because of their unique selfless and non-selective provision for the physical and pastoral needs of all (Stark, 2007). It is just such a retelling of this inclusivist provision of care by Australian military chaplains that moved a sceptical humanist historian to appreciate the contribution of faith-based chaplains, once the details of their service were chronicled accurately (Gladwin, 2013, p. ix).

## Faith-Based Chaplaincy

Given shifting societal religious and spiritual convictions, it is nevertheless timely to consider the current model of faith-based chaplaincy and what it offers organizations in the 21st Century. While there is a great deal of literature regarding the role of faith-based chaplains and many arguments which support the benefits of chaplaincy, this paper summarizes five main points with respect to the necessity for faith-based chaplains:

1. Chaplaincy and the Provision of Organizational Holistic Care
2. The Positive Role of Chaplains–but often unknown
3. Religious and Spiritual Well-being provided by Chaplaincy
4. Legacy of Religious Organizations maximized by Chaplaincy
5. Operational Advantage facilitated by Chaplaincy

### 1. Chaplaincy and Organizational Holistic Care

Over the centuries, faith-based chaplains have provided a substantial capability advantage for their organizations by providing holistic care. All employers are increasingly being obliged by legislation to provide for the physical and mental well-being of their employees (e.g., Safe Work Australia, 2022), a point that has come into sharp focus through regular reports of poor well-being outcomes for those working in areas that deal with high levels of trauma and death (Australian Senate Education & Employment References Committee, 2019; Commonwealth of Australia, 2022; Phoenix Australia & Canadian Centre of Excellence -PTSD, 2020; Sharp et al., 2020). That is to say, it is imperative for employers and leaders to ensure that the best possible holistic well-being support is provided for their personnel.

There is increasing evidence that contemporary best practice, that is truly holistic, involves spiritual care (also known as religious or pastoral care) (Cook & Moreira-Almeida, 2021; Royal Australian & New Zealand College of Psychiatrists, 2018; World Health Organization, 1998, 2002/2017). The ongoing need for spiritual and religious models of care have been increasingly made clear through research into moral injury (noted again later), which shows the importance of non-pathologized, spiritual and religious responses to moral distress (Carey & Hodgson, 2018; Carey et al., 2016; Koenig et al., 2023). And yet, spiritual care is only part of the continuum of care that faith-based chaplains provide.

#### Bio-Psycho-Social-Spiritual (BPPS)

The holistic care that faith-based chaplains bring is the manifestation of a bio-psycho-social-spiritual (BPSS) framework in which these domains are not mutually exclusive but interacting dimensions for every human being (Smith-MacDonald et al., 2018; Sulmasy, 2002). What this means in practice is that faith-based chaplains will utilize their training to provide professional assistance in matters pertaining to a person’s spiritual and/or religious beliefs. This care may include respectful listening, providing the perspective from ancient wisdom, pastoral counselling, or, as appropriate, praying with or for a person (Jankowski et al., 2011; Lewis, 2015). However, faith trained chaplains know that it is pointless praying for someone in need without providing for their physical needs and ensuring social support. Providing for the physical needs of a person and ensuring social support (of one kind or another) is a spiritual act that aids spiritual well-being; so physical and social support are also attended to by faith-based chaplains.

The initial act of the “first chaplain”, St Martin of Tours, was to provide for the physical needs of a soldier. In the contemporary world, chaplains may provide home cooked meals, a shoulder to cry on, transport, accommodation, or care for the person’s family. As one example of this, chaplains cooked and served hot soup to paramedics during winter as part of providing physical and emotional support while their ambulances were stuck in COVID-related delays at hospitals (Kolimar, 2022). Chaplains also attend to the social network of personnel including their families. In some jurisdictions faith-based chaplains are the only service who concurrently care for an organization’s active and former staff, as well as their families (Hartman, 2019). Faith-based chaplains are also trained in, and have engaged in providing psychological first aid, and referrals to mental health professionals as necessary (Tunks Leach et al., 2021).

The holistic view of the BPSS framework of chaplaincy work is combined with the historical flexibility of action that allows all the above services to converge to make faith-based chaplains like the “Swiss army knife” of staff care. It is in this context of staff well-being that faith-based chaplains have, do, and will provide holistic care for personnel (irrespective of their circumstances), and thus add value to organizations. The beneficial role of faith-based chaplains in providing spiritual and religious interventions as part of holistic care, increases all aspects of individual well-being, which has been regularly acknowledged over the last decade (Bonelli et al., 2012; Carey et al., 2016; Koenig, 2012; Lucchese & Koenig, 2013; Peng-Keller & Neuhold, 2020). Rather than providing just a professionalized transactional clinical encounter, faith-based chaplains incorporate many relational elements into their work such as inclusivity, supportiveness, and non-judgemental care for the staff and their family (Bugg, 2019).

### 2. The Positive Role of Chaplains—But Often Unknown

Chaplain is a term with which some may be unfamiliar. In certain contexts, there may also be resistance to utilizing faith-based chaplains, simply because some people are uncertain as to what a chaplaincy services involves (Clouzet, 2018). However, this is often overcome once people, religious or otherwise, do engage with faith-based chaplains. For example, members of the Australian public who have had previous experience with faith-based chaplains often report high levels of satisfaction following use of chaplaincy services. An independent report on school chaplaincy in Australia found that almost 9 in 10 school principals and 8 in 10 parents rated their National School Chaplaincy Program (NSCP) and their related activities extremely well in contributing to the emotional and social well-being of students (Kantar Public, 2016).

#### Hospital and Police Chaplaincy

Similarly, 65% of Australians who have previously received spiritual care while in hospital report an extremely or very positive experience, and 75% think this care should be readily available in all public hospitals (McCrindle Research Pty Ltd, 2021). Many emergency service organizations around Australia have also commenced or enlarged their chaplaincy teams in the last five years because they recognize the need to provide holistic care, and because faith-based chaplains are cost effective (Carey, 2014; Hausmann, 2004). The Australian Queensland Police Service have reported an almost exponential growth in the use of faith-based chaplains in the last 10 years (Baills, 2022). This suggests there is little evidence to indicate that faith-based chaplaincy services are not appreciated nor effective once engaged by even non-religious people–in fact quite the opposite: faith-based chaplains are sought after, effective and appreciated (Cafferky et al., 2017).

#### Paramedic Chaplaincy

The critical role of faith-based chaplains is also evident within paramedical services. Research on Australian paramedics reported that paramedics highly valued the support of faith-based chaplains, regardless of the paramedic’s personal spiritual or religious beliefs (Tunks Leach et al., 2021). Importantly, in the interviewed sample of 17 paramedics only three identified as religious, yet 16 of the 17 respondents thought faith-based chaplains added value to their organization even if they had not initially understood their role. The understanding that faith-based chaplains were appreciated was supported by chaplains themselves in follow-up research (Tunks Leach et al., 2022). It appeared, however, that the biggest barrier for personnel utilizing faith-based chaplains was uncertainty about their role, however, once used, faith-based chaplains were greatly appreciated for their care. This appreciation is reflected in the fact that in one of the largest Ambulance services in the world, reporting data indicated that chaplaincy is the most accessed staff support service, despite the presence of numerous secular alternatives (NSW Ambulance, 2021).

#### Military Chaplaincy

Similar levels of satisfaction are found within the Australian Defence Force (ADF). In the first ten months of 2021, approximately 170 full-time and 150 part-time ADF faith-based chaplains recorded their spiritual care activities (utilizing the World Health Organization codings); the total results revealed that faith-based chaplains implemented over 400,000 spiritual care interventions to assist military personnel and/or their families (Hynes, 2021). It is highly unlikely that the substantial utilization of ADF chaplaincy is because it is the only service available. In fact, faith-based chaplains are only a small part of the resources of the ADF; a force that also employs numerous secular supportive staff—namely over 300 full-time clinical health professionals, over 200 contracted mental health professionals, approximately a hundred psychologists and/or social workers, and with almost 1500 contracted external mental health and psychology support clinicians accessed via referrals.

To summarize it clearly; there currently exists a substantial number of secular services already available to ADF personnel, yet, despite the abundance of other resources, and despite declining religiosity, the faith-based chaplaincy model in the ADF seems to provide a popular and successful intervention program for staff–of all faiths or none. Of course, not everyone will want to engage with a chaplain, and they may not need to. However, the above figures indicate that a good number of Australian military personnel are comfortable in speaking to faith-based chaplains and are willing to seek their help (Hynes, 2021).

Further, the inclusion of faith-based chaplains makes for greater diversity which provides various perspectives for analysing issues (Bower, 2009). Faith-based chaplains thereby help to promote better decisions by commanders who may be blind to key pieces of information. Indeed, faith-based chaplains within the military are the key professionals who bring the spiritual/religious perspective, having been trained to help commanders understand more fully the spiritual aspect of people. Indeed, the spiritual/religious aspect is useful for a commander to consider with respect to those whom they command, and to understand the worldview of the enemy or the host nation with whom they are engaging in operational situations.

#### Accessing Chaplaincy

A number of barriers to accessing faith-based chaplains have been proposed. These include a lack of understanding regarding the role of chaplains (Boucher et al., 2018), or that some personnel only want a chaplain of their own faith position to attend to their spiritual needs (McCrindle Research Pty Ltd, 2021). However others report that personnel do not necessarily look for pastoral care from someone of their own faith, but rather the majority are happy to receive chaplaincy interventions irrespective of the chaplain’s own religious denomination (Kopacz et al., 2014). Finally some may experience a barrier because they may have a sense of abandonment by God (Jakucs, 2021), which ironically may be alleviated by the very chaplains they feel hesitant to engage.

Ever increasing antagonism from activist organizations stigmatizes those expressing religious beliefs (in supposedly inclusive secular organizations). This kind of antagonism itself has been suggested as a reason for the reduction in faith-based chaplaincy resourcing and operation (Bowlus, 2018). Some secularists insist that non-religious people do not wish to receive care from religious chaplains (Savage, 2015). However, as noted from the results of the largest international scoping review to date (Layson et al., 2022), such assertions are not supported by peer reviewed research–assertions which should be deemed inaccurate or at the very least somewhat misleading and “to be treated with caution” (Nolan, 2019).

The Layson et al. (2022) review, which summarized research that included 33 research studies involving a total of 19,366 participants, found there was no clear evidence that the spiritual orientation of military personnel affects their accessing of faith-based military chaplains. While there can be a degree of initial reluctance by some military personnel to engage with chaplains (often due to lack of knowledge regarding what chaplains do), nevertheless chaplains were considered a trusted and well appreciated service by those who had utilized chaplains (Nieuwsma et al., 2014; Roberts et al., 2018). Chaplains were also found to be important in helping personnel overcome any resistance to the use of psychologists (Besterman-Dahan et al., 2012). Even some who were opposed to using religious chaplains acknowledged the enduring trust of military personnel towards chaplains, and the large capability gap that would follow if chaplains were removed or exchanged (Surman, 2009).

#### Secularism

The aforementioned highlights that increasing secularism and declining religiosity has not resulted in falling satisfaction or decline in engagement with faith-based chaplains in Australia. In light of the ongoing use and popularity of faith-based chaplaincy, calls to remove faith-based chaplains because of falling religiosity levels (Hassanein, 2018; Hoglin, 2021b), can justifiably be argued to be invalid. Changes to faith-based chaplaincy provision cannot be vindicated merely by reference to changing census data, especially in the absence of any peer reviewed evidence to support such a move. What is more, as mentioned earlier, secular counsellors already exist within most critical workplaces in the form of psychologists, social workers, and other mental health providers—whereas faith-based chaplaincy ensures a truly holistic bio-psycho-social-spiritual approach to care. This care complements existing secular services ensuring a balanced approach for the health and well-being of all personnel (both religious and non-religious); a point which is noted again later in this paper. Some secularists, however, remain strongly opposed to the institutional ministry of faith-based chaplaincy within public institutions. To this point, it is well worth noting the comments of Nolan (2019) in response to Savage’s (2019) textbook on “*Non-Religious Pastoral Care*”:

Where the book fails, however, is in its perpetuation of the idea that chaplaincy is fixated on religion, in this case the having or not having of it. The mistake of this book is that it characterizes “chaplaincy” care in terms of “religious care”, ignoring its advocacy work, its contribution to equality and diversity, and its fundamental willingness to be present to all forms of human suffering (in many cases 24 hours a day, 7 days per week). Even where chaplains have followed a strongly sacramental model of chaplaincy … their concern has never been limited to providing religious care; rather chaplains have served people in the exigencies of their human condition, through birth to death and all the intervening transitions of life (Nolan, 2019, p. 99).

Further to Nolan’s point, the editors of the *Journal of Religion and Health* summarize, that substantial research relating to the efficacy of faith-based chaplaincy is “…thriving and proving the benefits of chaplaincy pastoral and spiritual care” (Carey et al., 2023). They go on to state: “The outcomes of this chaplaincy research challenges the rationalist philosophies and sabotaging activities of those secularists who deliberately or subtly discriminate against people needing or wanting the provision of pastoral and spiritual care interventions—particularly for people with religious affiliations—whether they are in hospitals, aged care facilities, welfare, mental health care, educational institutions, refugee centres, correctional facilities, the military, police force, firefighter or paramedical services” (Carey et al., 2023, pp. 1–2).

### 3. Religious and Spiritual Well-Being Provided by Chaplains

Secular humanist organizations often neglect to mention that many secular mental health well-being options already exist alongside faith-based chaplains (as noted earlier). Indeed, it has been argued that personnel are actually serviced much better and more efficiently by pastorally trained and qualified faith-based chaplains who work collaboratively with accredited secular mental health professionals (Ames et al., 2021; Carey & Hodgson, 2018). Of course, the chaplain’s primary role is not to provide mental healthcare per se, however, faith-based chaplains do regularly provide psychological first aid, or become facilitators for clients to gain a referral for appropriate mental healthcare. Predominantly faith-based chaplains take a holistic view of the needs of the person they are assisting during the provision of spiritual care. As also mentioned earlier, chaplains begin from a relational stance that transcends a focus on clinical interventions.

Faith-based chaplains are motivated by a calling to dependably provide their services, largely on call 24 h. A chaplain’s wide scope of practice is advantageous for many reasons. When a chaplain encounters a staff member, rarely are the staff member’s needs contained to one domain such as mental, emotional, spiritual, or practical. This reflects the interacting domains of the BPSS model of the human. Faith-based chaplains move between the various forms of intervention of providing religious/spiritual assessments, religious/spiritual counselling, guidance, and education, various religious and/or spiritual rituals, or by providing spiritual support through attending to physical and practical needs.

Additionally, as noted earlier, while faith-based chaplains are not mental health professionals, they are allied health providers that help personnel to deal with spiritual matters (Carey et al., 2018). Indeed, chaplains have been noted to be an important pathway to mental health services, and even help personnel overcome barriers to mental health care (Besterman-Dahan et al., 2012; Nazarov et al., 2020; Nieuwsma et al., 2014). Faith-based chaplains can offer an avenue of support that is usually considered by personnel as being far more confidential than that provided by a mental health professional. Despite possible criticism of the tight confidentiality faith-based chaplains maintain, without it “fewer service members would seek assistance for their problems, resulting in higher rates of suicide, substance use and mental health disorders, domestic violence, and other problems” (Bulling et al., 2013, p. 561). Faith-based chaplains are often more trusted with regard to complex moral conundrums and the various religious and spiritual issues that can be associated with mental health concerns (Carey et al., 2015). Nevertheless, several chaplaincy researchers affirm that collaboration between faith-based chaplains and mental healthcare providers is recommended as a mutually enhancing relationship to successfully return service personnel to a good state of health (Seddon et al., 2011).

The chaplain’s main role is to provide spiritual, pastoral, and religious care through a variety of means (e.g., counselling, guidance, education, support, and rituals). Human spirituality is not co-terminus with religion, nor is it synonymous with social sciences. Those with religious training, study spirituality both generally and specifically to be able to discern spiritual and religious matters. This is not the case, however, with all health science and social science training. Very few, if any, medicine, psychology, nursing, paramedicine, or social work qualifications give even the briefest attention to spiritual matters as part of their professional training. Conversely subjects that entail the psychological elements of distress are usually present in chaplaincy tertiary courses, as well as clinical pastoral education—giving chaplains that additional knowledge and training advantage.

#### Spirituality

Spirituality has been defined as “a dynamic and intrinsic aspect of humanity through which persons seek ultimate meaning, purpose, and transcendence, and experience relationship to self, family, others, community, society, nature, and the significant or sacred. Spirituality is expressed through beliefs, values, traditions, and practices” (Puchalski et al., 2014). Spiritual well-being can be measured as being independent of specific religious beliefs. However, the more generic nature of spirituality defined by Puchalski et al. means that those who hold particular religious beliefs about God may not be well serviced by spiritual carers who omit religious and theological understandings of well-being. Attending to transcendent themes and beliefs is an essential part of holistic care that promotes good health (Koenig, 2012) and is integral to the faith-based chaplain’s training. However, transcendence, religion, and faith, are largely rejected by secular humanist organizations, with many even rejecting “anything remotely spiritual” (Francis, 2021, p. x). Despite this, the same organizations claim that secular humanists can somehow attend to transcendent or spiritual beliefs, and some attempt to do so without direct training in these fields—which raises issues of professional and personal integrity.

The relational emphasis of the faith-based chaplain often challenges the barriers created by clinical isolationism, enabling faith-based chaplains to become a companion along the way, a trusted confidante, a wise counsellor, or can even be considered what one might call an “expert companion” (Tedeschi & Calhoun, 2013) or a “professional friend” (Trygstad, 1986). In situations of moral conflict chaplains can act as a benevolent moral authority (Litz et al., 2009) where acceptance and forgiveness can be found. Chaplains of faith practically implement a transcendent perspective that is often needed at times of distress, grief or when one’s mortality is clear and close. The presence of military faith-based chaplains on the battlefield, for example, can raise the morale of soldiers (Seddon et al., 2011), helping to provide protection against mental breakdown.

Indeed, a large body of evidence reports that ignoring the spiritual and religious elements of life can have a significantly detrimental effect on the identity of and meaning of life for personnel that can lead to existential distress, and eventually to negative impacts on both physical and mental well-being if not addressed at a spiritual level (Amato et al., 2017; Bonelli et al., 2012; Boucher et al., 2018; Bowlus, 2018; Dhar et al., 2011; Hamlin-Glover, 2011; Koenig, 2009; Pargament et al., 2013; Rogers, 2020; Smigelsky et al., 2020; Unterrainer et al., 2010). While it could be argued that spiritual guidance could be sourced outside of the workplace, when spiritual care occurs from those “on the inside” (i.e. those who have awareness of the organizational culture) it is received much more readily (Tunks Leach et al., 2020, 2021).

Chaplains have long been the subject matter experts (SME) in spiritual and religious struggles, including for some non-religious people who may maintain a “religious residue” or vicarious religion long after having de-identified from any religion (Davie, 2015; Van Tongeren et al., 2021). The residual or vicarious nature of religion means that many non-religious people have a tacitly held framework of just world beliefs and moral expectations that have often been formed through a religious cultural lens, despite their outward rejection of religious labels. Faith-based chaplains have the training needed to help those who may not appreciate their latent moral convictions, to re-examine these when distress arises. Religious chaplains can help articulate to personnel how their faith stance may be causing harm and point them to an alternate theological or worldview perspective, as well as to other support services. Chaplains often discuss forgiveness, restoration, and hope, that can reduce distress; and because they have the religious knowledge and skills, faith-based chaplains can even provide specific religious healing rituals and prayers—if and when it is requested or appropriate.

#### Moral injury

The spiritual elements of distress are particularly important when considering issues to do with death, trauma, betrayal, and violation of just world beliefs—regular occurrences in critical environments—that can manifest as moral injury. Moral injury is increasingly being recognized as a problematic syndrome among military populations (Koenig & Al Zaben, 2021), health care workers (Williamson et al., 2022), and first responders (Mordeno et al., 2022; Smith-MacDonald et al., 2021). While no consensus definition yet exists, moral injury is defined by the Australian Defence Force as:

a trauma-related syndrome caused by the physical, psychological, social and spiritual impact of grievous moral transgressions, or violations, of an individual’s deeply held moral beliefs and/or ethical standards due to: (i) an individual perpetrating, failing to prevent, bearing witness to, or learning about inhumane acts which result in the pain, suffering or death of others, and which fundamentally challenges the moral integrity of an individual, organization or community, and/or (ii) the subsequent experience and feelings of utter betrayal of what is right caused by trusted individuals who hold legitimate authority (ADF, 2021).

The research on moral injury consistently reports that spiritual and/or religious themes often arise, and an increasing number of the latest therapeutic regimes recommend against purely psychological interventions. Instead, they recommend the collaboration between a psychologist with a religiously trained chaplain to deal with trauma to the soul (Ames et al., 2021; Antal et al., 2019; Carey & Hodgson, 2018; Pernicano et al., 2022; Pyne et al., 2019; Rudolfsson & Milstein, 2019; Wortmann et al., 2021). This collaboration is required because a moral injury is not like a physical injury that simply mends with clinical treatment. It requires the exploration of underlying beliefs and spiritual and religious coping strategies. Maintaining a well-trained group of faith-based chaplains, who are conversant with the often tacitly held religious beliefs of both the religious and non-religious, is crucial to responding to moral injury. This chaplaincy capacity helps to sustain a “spiritual readiness” so that, for example, military personnel are warrior-ready for the “battles” and the consequences that lay ahead (Koenig et al., 2022).

### 4. The Legacy of Religious Organizations Maximized by Chaplaincy

Faith-based chaplaincy connections with established religious organizations and charities often allow chaplains to source a diverse range of services for personnel from local and national faith-based charities. Additionally, faith-based chaplains are able to draw on a rich theological and philosophical history that spans millennia of thinking and benevolent action related to life, death, and injustice. However, since the World Trade Centre attacks in 2001, it has become somewhat of a cliché, that religion seems to do nothing but harm society (Dawkins & Ward, 2006; Hitchens, 2008). Indeed, this argument has been corralled in the hope to remove faith-based chaplains by claiming, “religion remains a well-documented source of cultural division” (Hoglin, 2021a, 2021b). However, the claim that religion’s impact is largely divisive deserves closer inspection.

It is truer to say that bad religion and poor religious coping strategies divide and harm, while good religion and good religious coping strategies bring healing and unity (O'Connor & van Dijk, 2012; Park et al., 2018; Stark, 2004). There is a positive aspect of religion that is less commonly acknowledged by the secularists mentioned above. It is this positive legacy of charitable community action and scholarly thought that chaplains are able to leverage for the good of all personnel—irrespective of cultural or religious beliefs.

#### Charity and Unity

While undoubtedly religion has had historical periods of division, religion is also associated with prosocial behaviours that increase unity, charity, and peace. Figures such as Desmond Tutu (1931–2021), William Wilberforce (1759–1833), John Newton (1725–1807), The Dalai Lama (1935–), Maulana Wahiduddin Khan (1925–2021), and countless faith-based charities attest to this. For example, four of the top five charities in Australia are Christian organizations (Payne, 2014) and in a 2021 survey, 76 per cent Australians agreed that churches in their local area were making a positive difference to their community during the COVID-19 pandemic (McCrindle & Renton, 2021). Similarly, scholars of religion have recognized this impact. While acknowledging that religious factors have contributed to political divisions in the past, several academics have written that these tensions are minimal compared to the ongoing frictions caused by secular individualism (Huntington, 1993; Gardels, 2013; Jupp, 2011). On the extreme end of the secular-atheist political spectrum characters such as Karl Marx (1818–1883), Joseph Stalin (1878–1953), Mao Zedong (1893–1976) and Pol Pot (1925–1998), have all contributed to extensive anti-religion violence.

When a chaplain operates in their role as a spiritual care provider, they follow a long tradition of care for the individual and can call upon an extensive and well-established network of resources from their faith community and local charity networks to provide for the needs of personnel. In Australia, faith-based chaplains can leverage the services of organisations such as Anglicare, BaptCare, Mission Australia, Muslim Aid Australia, Salvation Army, Sikh Volunteers Australia, St Vincent de Paul, Tzu Chi Australia, and UnitingCare. Additionally, local churches, mosques, and temples, all provide a diverse range of services and opportunities for social connections which faith-based chaplains can engage for the good of staff, and to the advantage of their organizations.

#### Religious Thought

Religious thought, for example, on the correct conduct during war, has a long and important role with regard to morality. *Just war theory* provides an important framework for deciding whether the waging of any war itself is just and whether or not the specific action a soldier undertakes is just. Historically, others recount the positive impact of religious and moral lessons for troops during training periods in Egypt during WWII and the Japanese Occupation Force post WWII, in order to deter soldiers from abusive sexual relationships with local women (Davidson, 2021; Gladwin, 2013). Killing another human is counter to the moral framework of many people and can lead to moral distress (Burkman et al., 2022; Grossman, 2009). To lose a framework that helps make sense of war, may leave military forces in a position where soldiers could either struggle to justify their actions or kill without conscience. Faith-based chaplains leverage this long tradition of thought to help commanders to consider the moral and ethical implications of their directives and help soldiers to find solace when they feel they have crossed their own moral lines (Hodgson et al., 2021, 2022). It is the fusing of just war theory and the notions of grace and forgiveness within many religions that can bring some of the most profound healing from the invisible wounds of war (see earlier–“moral injury”; Koenig et al., 2022).

### 5. Operational Advantage Facilitated by Chaplaincy

Finally, it is important to come back to the reality of the overwhelmingly religious nature of our world. In light of this, many of the secular arguments against faith-based chaplains, that arise in Western contexts, contain hints of a new form of secular colonialism. Many organizations that provide critical services operate in dangerous places and among communities where religion is important. These critical services may be provided in war zones, or to disadvantaged communities, or non-Western populations, among whom levels of religiosity remain high. For example, Australian military deployments to Timor, the Solomon Islands, Tonga, and the like, have all been enhanced by the engagement of faith-based chaplains with the local community (March, 2022; Trembath, 2022).

Faith-based chaplains are grounded in religious frameworks and can offer important perspectives on the motivations of conflict and strategies for peaceful resolution. Interestingly the world’s (supposedly) least religious nation, China, has been experiencing a rapid growth in religion (Carey et al., 2022). The very high level of “no-religion” affiliation in countries like China is projected to fall by 11 per cent by 2060, with religiosity expected to increase. This changing religious landscape may mean that an agency with exposure to the world’s most populated nation will have a diminished impact without the capability to engage with a shifting Chinese culture (Huang & Hu, 2019). Faith-based chaplains can provide important connections with local community and religious leaders which benefit the community, can help calm tensions, and encourage positive and peaceful expression of religious beliefs.

## Conclusion and Recommendations

The factors above suggest that organizations which terminate or reduce their faith-based chaplaincy resources, will lose substantial capability to holistically care for the general well-being of its personnel, particularly in areas of speciality (e.g., spiritual and religious coping, moral injury, intercultural/inter-religious networking).

Without faith-based chaplains, organizations may also be more constrained in multi-cultural recruitment or engagement, and unable to fully understand the underlying motivations of those it is meant to protect or even engages. While faith-based chaplains do not require, nor seek, religious affiliation from those they help, faith-based chaplains do understand with a depth of insight the issues around identity, meaning, and purpose that face religious and non-religious people alike. To conclude, we make the following recommendations for organizations to consider regarding faith-based chaplaincy.

First, organizations should continue to pursue those of diverse religious background to serve as faith-based chaplains. Pursuing additional faith-based chaplains of varying religious backgrounds will further enhance the diversity and inclusivity of organizations through their very presence. A varied faith-based chaplain capability will encourage personnel of varying faiths who often have non-European backgrounds.

Second, the role and capability of faith-based chaplains should be clarified for all staff, who may have an initial reservation about engaging with a significant well-being capability asset which they may not understand, yet which could be of tremendous benefit to them.

Third, as there is no scholarly or credible evidence that the religious nature of faith-based chaplaincy provides any significant barrier to the utility of faith-based chaplaincy, nor that it lacks efficacy (Layson et al., 2022), it would run contrary to existing evidence to reduce a well-established asset that is trusted, extensively used, and which has a long-standing tradition of service through many critical events over decades with a proven benefit (Gladwin, 2013; Shay, 2014; Strong, 2012).

Clearly, efforts should continue to allow for better interdisciplinary collaboration between faith-based chaplains and other staff well-being options such as psychologists and social workers—indeed organizations should increase the services of allied health professionals as well as increasing faith-based chaplains enabling greater collaboration and to ensure a holistic approach—an approach which chaplaincy has long provided, and continues to provide, as a capability advantage for the benefit of organizations and for their personnel.
