# Correction: The Impact of Faith‑Based Pastoral Care in Decreasingly Religious Contexts: The Australian Chaplaincy Advantage in Critical Environments

**DOI:** 10.1007/s10943-023-01829-0

**Published:** 2023-05-17

**Authors:** Mark D. Layson, Lindsay. B. Carey, Megan C. Best

**Affiliations:** 1 Faculty of Arts and Education, St Mark’s National Theological Centre, Charles Sturt University, Canberra, ACT, NSW Australia; 2 Palliative Care Unit, La Trobe University, Melbourne, VIC Australia; 3 Institute for Ethics and Society, University of Notre Dame, Sydney, Australia; 4 Centre for Spirituality, Theology and Health, Duke University, North Carolina, USA; 5 School of Medicine, University of Sydney, New South Wales, Australia

**Correction to: Journal of Religion and Health (2023) 62:1491–1512** **https://doi.org/10.1007/s10943-023-01791-x**

The article “The Impact of Faith-Based Pastoral Care in Decreasingly Religious Contexts: The Australian Chaplaincy Advantage in Critical Environments”, written by Mark D. Layson, Lindsay. B. Carey and Megan C. Best, was originally published Online First without Open Access. After publication in volume 62, issue 3, page 1491–1512 the author decided to opt for Open Choice and to make the article an Open Access publication. Therefore, the copyright of the article has been changed to © The Author(s) 2023 and the article is forthwith distributed under the terms of the Creative Commons Attribution 4.0 International License, which permits use, sharing, adaptation, distribution and reproduction in any medium or format, as long as you give appropriate credit to the original author(s) and the source, provide a link to the Creative Commons licence, and indicate if changes were made. The images or other third party material in this article are included in the article’s Creative Commons licence, unless indicated otherwise in a credit line to the material. If material is not included in the article’s Creative Commons licence and your intended use is not permitted by statutory regulation or exceeds the permitted use, you will need to obtain permission directly from the copyright holder. To view a copy of this licence, visit http://creativecommons.org/licenses/by/4.0.

